# A quality assurance protocol for reliable and reproducible multi-TI arterial spin labeling perfusion imaging in rat livers

**DOI:** 10.1007/s10334-024-01223-1

**Published:** 2025-01-04

**Authors:** Wan-Ting Zhao, Karl-Heinz Herrmann, Weiwei Wei, Martin Krämer, Uta Dahmen, Jürgen R. Reichenbach

**Affiliations:** 1 Medical Physics Group, Institute of Diagnostic and Interventional Radiology, Jena University Hospital, Friedrich Schiller University Jena, Jena, Germany; 2 Department of General, Visceral and Vascular Surgery, Experimental Transplantation Surgery, Jena University Hospital, Jena, Germany; 3 Institute of Diagnostic and Interventional Radiology, Jena University Hospital, Friedrich-Schiller University Jena, Jena, Germany

**Keywords:** Perfusion, Arterial spin labeling, Liver, Reproducibility, Preclinical study

## Abstract

**Objective:**

To establish an arterial spin labeling (ASL) protocol for rat livers that improves data reliability and reproducibility for perfusion quantification.

**Methods:**

This study used respiratory-gated, single-slice, FAIR-based ASL imaging with multiple inversion times (TI) in rat livers. Quality assurance measures included: (1) introduction of mechanical ventilation to ensure consistent respiratory cycles by controlling the respiratory rate (45 bpm), tidal volume (10 ml/kg), and inspiration: expiration ratio (I:E ratio, 1:2), (2) optimization of the trigger window for consistent trigger points, and (3) use of fit residual map and coefficient of variance as metrics to assess data quality. We compared image quality, perfusion maps, and fit residual maps between mechanically ventilated and non-ventilated animals, as well as repeated ASL measurements (session = 4 per animal) in two mechanically ventilated animals.

**Results:**

Perfusion measurements over multiple sessions in mechanically ventilated rats exhibited low perfusion data variability and high reproducibility both within and between liver lobes. Image quality and perfusion maps were significantly improved in mechanically ventilated animals compared to non-ventilated animals.

**Discussion:**

The implementation of mechanical ventilation and optimized quality assurance protocols enhanced the reliability and reproducibility of FAIR-based multi-TI-ASL imaging in rat livers. Our findings demonstrate these measures as a robust approach for achieving consistent liver perfusion quantification in preclinical settings.

**Supplementary Information:**

The online version contains supplementary material available at 10.1007/s10334-024-01223-1.

## Introduction

Magnetic resonance imaging (MRI) is a powerful non-invasive modality offering exceptional soft-tissue contrast, enabling detailed visualization of organs and tissues while tracking morphological and functional changes over time. This versatility makes MRI indispensable for longitudinal studies of disease progression and therapeutic efficacy. Among its many applications, organ perfusion measurement is a cornerstone for evaluating tissue health and function. In liver research, accurate perfusion assessment is essential not only for diagnosing and managing hepatic conditions but also for preclinical studies involving animal models. Advancing MRI-based methodologies for preclinical perfusion assessments is therefore critical to improving the precision and reliability of liver perfusion measurements, ultimately enhancing experimental models and their translational value.

Liver perfusion can be assessed using MRI through various methods, including dynamic contrast-enhanced MRI (DCE-MRI) [1], dynamic susceptibility contrast MRI (DSC-MRI) [2], and arterial spin labeling (ASL). Although contrast-enhanced imaging techniques are highly effective for detecting perfusion changes, their application in patients is often constrained by the risks associated with contrast media [3]. In preclinical research, contrast agents can be used under controlled conditions; however, their repeated administration in longitudinal animal studies raises concerns about cumulative physiological effects and animal welfare. This limitation highlights ASL as the most suitable tool for longitudinal studies, offering a non-invasive alternative without the need for contrast agents.

ASL-MRI was initially developed for neurological applications to examine cerebral perfusion [4, 5] but has since been adapted for use in various application fields, including tumor differentiation [6], kidney function assessment [7, 8], and myocardial blood flow measurement [9]. However, its application to liver imaging introduces unique challenges. First, the liver’s dual blood supply via the portal vein and hepatic artery poses substantial difficulties as these two sources of blood flow differ in pressure and volume, complicating the modeling of different compartments and their interactions. Second, respiratory motion further challenges the precision of liver ASL-MRI. Efforts to mitigate its effects have been made using tailored sequences, advanced reconstruction techniques, and additional instruments [10]. However, these solutions remain limited and are implemented by only a small number of research groups [9–11].

The basic principle of ASL-MRI is the use of magnetically labeled proton spins in the arterial blood supply, which feeds the tissue or organ of interest as an endogenous tracer [12]. Labeling is achieved by modifying the longitudinal magnetization, usually by inversion. After a controlled delay, the inversion time (TI), to allow for the distribution of these labeled protons throughout the vasculature and tissues of the organ, an image is acquired. The difference between this labeled image and a second control image (with unlabeled arterial blood) then provides a measure of the blood flow to the organ, i.e., a perfusion-weighted signal [4, 13].

**Fig. 1:**
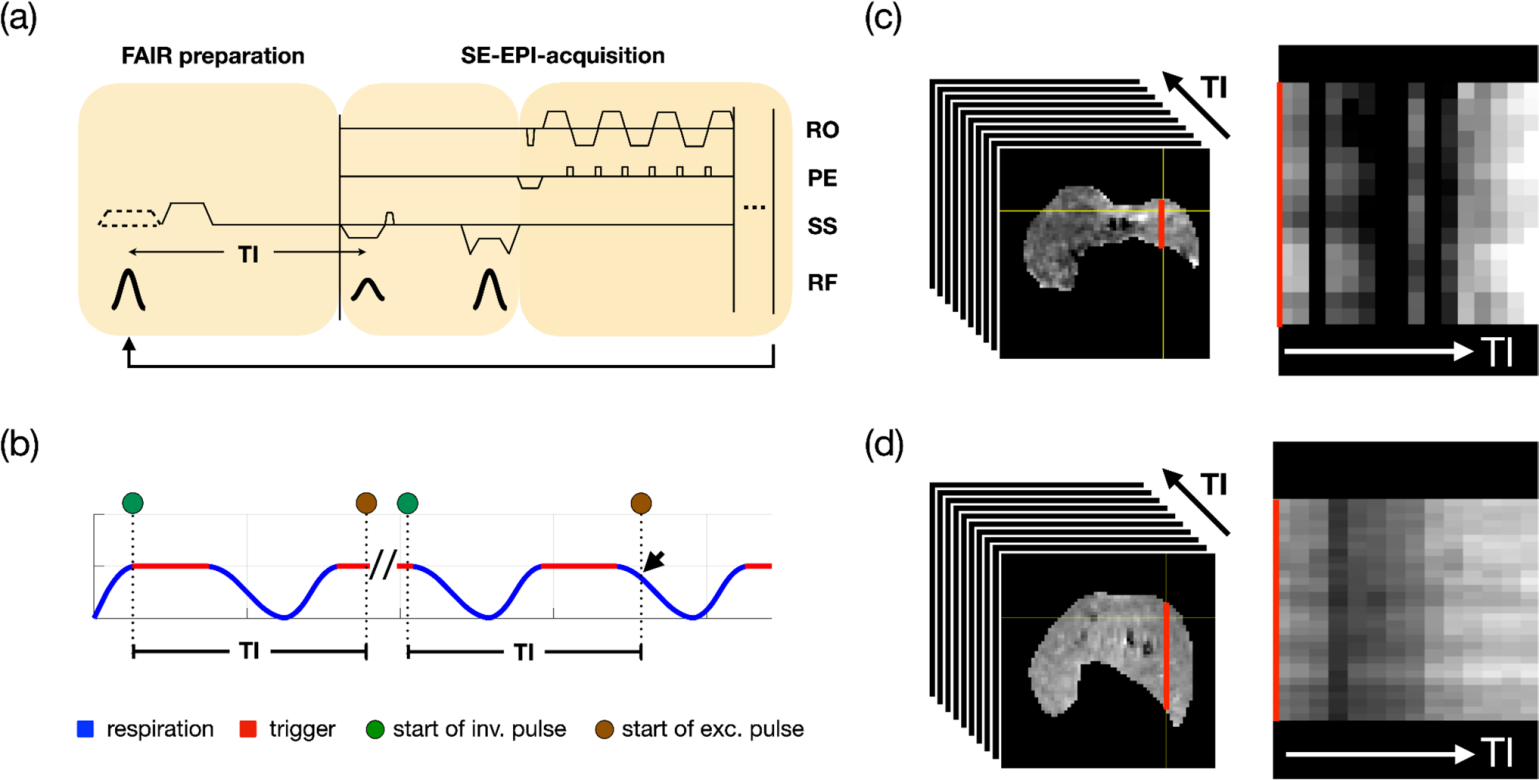
**a** Diagram of a FAIR-SE-EPI sequence. The sequence is triggered and starts with an adiabatic 180° inversion pulse, followed by a TI interval during which the longitudinal magnetization recovers. After the TI, the slice is imaged with an SE-EPI-acquisition module followed by a constant recovery period of 10 s to ensure complete relaxation. This sequence module is repeated alternately with slice-selective (label) and global inversion (control), i.e., with or without applying slice-selective gradients. For multi-TI FAIR imaging, this FAIR sequence is repeated with different inversion delays TI. **b** The diagram highlights the importance of correct triggering in an FAIR sequence with a fixed inversion time (TI) and extended trigger windows (indicated by red lines). On the left, both the inversion and slice imaging occur within end-exhalation phase, minimizing motion-induced mismatch artifacts. In contrast, the right part illustrates a scenario where the inversion is triggered at the end of the extended trigger window, causing the subsequent slice imaging to align with the inspiration phase of the respiratory cycle. This results in imaging of a shifted slice in the liver. This is particularly important for the slice-selective inversion, where the temporally separated inversion and imaging slices have to match. **c** and **d** Controlling respiration significantly enhances image quality. A comparison of image profiles (red lines) in FAIR perfusion datasets with multiple TIs under free breathing (**c**) and mechanical ventilation (**d**) demonstrates the advantages of mechanical ventilation. The data show a clear reduction in profile artifacts throughout the TI series when mechanical ventilation is used, leading to more stable and reliable perfusion results

While continuous ASL (CASL) involves the continuous delivery of a radio frequency (RF) pulse in the presence of a gradient field to label incoming blood in a plane upstream to the imaging slice, pulsed ASL (PASL) employs short inversion RF pulses (5–20 ms) to intermittently label blood in a thick slab with high labeling efficiency [14]. Flow-sensitive alternating inversion recovery (FAIR) [15] is a variant of PASL. It begins the acquisition of the label image with the application of a slice-selective inversion pulse to label the slice of interest, followed by an inversion delay TI before image acquisition by a readout module such as EPI (Fig. 1a). The control image is acquired after applying a non-selective inversion pulse that inverts the spins throughout the sensitive volume of the RF coil. Systematically performing the acquisition protocol with multiple inversion times (multi-TI FAIR) enables the sampling of inverted magnetization recovery kinetics, thereby allowing accurate quantification of two T_1_ maps, which differ solely due to the applied labeling effect. As a result, it enables the creation of a perfusion map that minimizes the influence of confounding tissue conditions, including liver steatosis [16], fibrosis [17], and cirrhosis [18], which can cause T_1_ and T_2_ changes in the tissue.

When performing liver perfusion experiments in animals, it is essential to precisely control the timing of both the inversion pulse and the excitation pulse initiating imaging during the end-expiratory phase. Failure to do so can result in motion artifacts, such as blurring and ghosting, which can affect image quality and lead to misalignment of images acquired with different TIs. The challenge is to ensure that the tagged slice with magnetization inverted by the inversion pulse (Fig. 1b, green) is in the same position during image acquisition (Fig. 1b, brown), especially in the case of selective labeling with a long inversion time (TI). If respiratory motion shifts the liver during this waiting period, the inverted slice will not be aligned correctly with the imaging slice, leading to inaccurate perfusion results. A long (prospective) trigger window is typically used to maximize scan efficiency. However, with this approach, the inversion pulse can start at any point during the relatively motion-free exhalation state, which leads to variability in the timing of the inversion and excitation pulses in relation to the breathing phase. For example, for a given TI, the start of inversion at the beginning or end of a long trigger window can lead to different perfusion results (Fig. 1b). In the first scenario, a trigger initiates the inversion pulse early in the trigger window, resulting in inversion and image acquisition in a motion-free phase (Fig. 1b, first TI). In contrast, if the trigger initiates the inversion pulse near the end of the window, the excitation pulse may coincide with the strong motion during inspiration (Fig. 1b, second TI, arrow), leading to imaging of a different liver slice than the one prepared by the inversion pulse and resulting in motion-induced artifacts and corrupted results. Such displacements of the liver can be quite severe, and respiratory-induced diaphragm shifts of up to 2 mm in the superior–inferior direction have been reported in rats [19]. These shifts can be even larger (up to 3.5 mm, own observations) during deep exhalation, a reflex that can occasionally be observed in free-breathing animals subjected to isoflurane inhalation. In our case, the displacement in a free-breathing rat is ~ 17% of the in-plane size of the liver, which is substantial and not negligible.

Therefore, our study aimed to develop a simple and effective quality assurance protocol to reduce the effects of respiratory modulation during multi-TI-FAIR perfusion measurements in rat livers. Our approach involves three measures: (1) implementing mechanical ventilation (MV) to ensure steady and predictable respiration, (2) optimizing the timing of inversion delays (TIs) to guarantee that both the inversion pulse and image acquisition occur during motion-free phases, and (3) utilizing fitting residuals during post-processing to assess and visualize data quality. We compared the calculated perfusion maps and data quality obtained under mechanical ventilation with those acquired in free-breathing (i.e., non-mechanical ventilation, NMV) animals. The comparison included three scenarios: NMV, MV, and MV where the voxels with pulsation were excluded. To demonstrate the effectiveness of this protocol, we applied the optimized methodology to two rats, each undergoing four ASL-MRI sessions. The resulting perfusion measurements showed low variability across different liver lobes, highlighting the reproducibility of the method.

## Materials and methods

### Animal experiments

The study was approved by the relevant institutional animal care and use committee (IACUC) and conducted under the authorization of the local governmental authority, the Office of Food Safety and Consumer Protection (Thüringer Landesamt für Verbraucherschutz, Bad Langensalza, Germany; local registration numbers: UKJ-17-106, UKJ-22-006). Animals were housed under controlled conditions, including a 12/12 h light–dark cycle, a temperature of 22 ± 2 °C, and a humidity level of 50 ± 10%. They had access to a pellet diet and water ad libitum. Four adult male rats with a mean body weight of 519 ± 157 g were included, all of which underwent MRI measurements. Two animals were subjected to a single session of non-mechanical ventilation (NMV), while the other two underwent four sessions of mechanical ventilation (MV).

### Mechanical ventilation and capnography

Before commencing work with animals, the ventilator (MRI-1 Ventilator, CWE Inc., Ardmore, PA, USA) was tested for tidal volume by attaching it to a small balloon. The capnography (MicroCapStar, CWE Inc., Ardmore, PA, USA) was calibrated to a 5% readout using clinical carbogen gas (5% CO_2_, 95% O_2_).

For the in vivo experiments, the rats were anesthetized with 2.5% isoflurane for 10 min using a 0.7/0.3 air/O_2_ mixture. After confirming the absence of response to interdigital noxious stimuli, the NMV animals were immediately transferred to the scanner cradle, where gas anesthesia was maintained via a nose cone. The respiration rate was carefully controlled within the 45–50 bpm range by adjusting the isoflurane concentration, typically 2.5–3%. For the MV animals, once the interdigital reflex was absent, the animals were placed on a 3D-printed tilting work stand with their front teeth secured [20] (inclined plate with strung wire for the teeth) to ensure a slightly extended neck, facilitating smooth intubation. Under continuous isoflurane anesthesia, the animals were orotracheally intubated using the plastic parts of a 14-gauge catheter [21], assisted by a soft guide wire and an otoscope [22] (Rat intubation pack, Hallowell, EMC, MA, USA). The intubated animals were then transferred to the MRI system’s cradle and mechanically ventilated at a rate of 45 bpm with a tidal volume (V_T_) of 10 ml/kg [21]. The inspiration:expiration ratio (I:E ratio) was set at 1:2 [23]. A continuous side-stream end-tidal CO_2_ (EtCO_2_) was measured by a T-branch connection for gas sampling for capnography.

To optimize ventilation and prevent spontaneous respiratory motion without the need for muscle relaxants, isoflurane concentration was maintained at 3%. End-tidal CO₂ (EtCO₂) levels were kept between 4.5% and 5.4% (34–41 mmHg), indicating normocapnia throughout the measurements. To further enhance ventilation efficiency, the tubing connecting the remotely operated valves for inhalation and exhalation was refined (see also Supplementary Figure S3). Dead volume was minimized to accommodate the capnography sampling branch, and the tubes between the Y-connector and the valves were shortened to 40 mm to reduce exhalation resistance.

### MRI procedure

All in vivo studies were conducted at 9.4 T using a BioSpec 94/20 small animal MRI system (Bruker BioSpin, Ettlingen, Germany) equipped with a 0.7 T/m gradient system (BGS-12, Bruker) and ParaVision software version 6.0.1. Imaging was performed using a rat whole-body quadrature volume coil with an inner diameter of 86 mm (Bruker BioSpin, Ettlingen, Germany). Each animal was positioned supine on a rat cradle, with body temperature continuously monitored via a rectal probe and maintained at 37 ± 2 °C using a feedback-controlled warm-water heating system (SA Instruments Inc., Stony Brook, NY, USA). In addition to pulse monitoring, respiration in both MV and NMV experiments was monitored with a pneumatic pillow sensor attached to the animal’s side abdomen, connected to a pressure transducer (SA Instruments, Inc., USA). For anatomical reference and geometric planning, as well as to determine the perfusion slice, a respiratory-gated, T_2_-weighted, 3D variable flip angle rapid acquisition with relaxation enhancement (varFlipRARE) sequence [24] was employed. This in-house developed sequence uses short, non-selective block pulses with variable flip angles to lengthen the effective T_2_, allowing longer echo trains. After three calculated initial flip angles bringing the spin state into a pseudo steady state for 25°, the flip angles follow three linear ramps: 25° to 45° in 41 steps, 46° to 110° in 31 steps, and, finally, 110° to 180° in 25 steps [24–26]. Imaging parameters were as follows: TR/ΔTE = 1000/1.97 ms, nominal TE = 38 ms, RARE factor = 100, isotropic resolution = (350 μm)^3^, bandwidth = 250 kHz; with respiratory synchronization, the effective TR was 1.33 s at 45 bpm, resulting in a total acquisition time (TA) of approximately 5 min and 20 s.

### Liver perfusion protocol

**Fig. 2:**
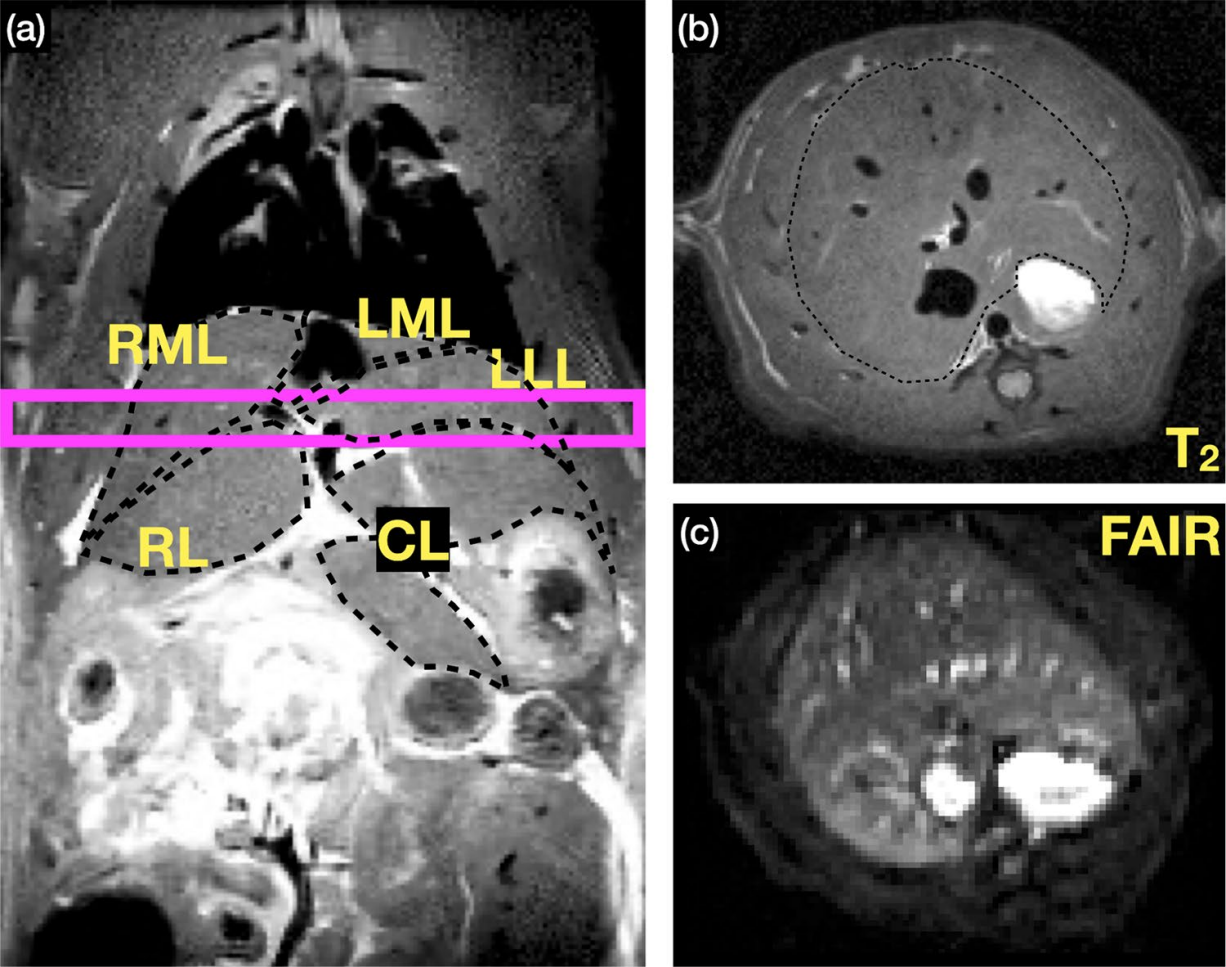
The planning of the single-slice FAIR ASL-MRI in the rat liver. **a** A 2 mm axial slice (magenta) was planned on a 3D T_2_w image to maximize the in-slice coverage of the rat liver lobes. **b** The resulting T_2_w axial slice and **c** the corresponding FAIR image (exampled in TI = 1500 ms). The segmentation of the liver lobes was based on the calculated T_1_ map and the contrast of the peritoneal fluid versus liver tissue to depict the rim between different lobes. Because the signal nulling and relaxation are faster in the tissue than in the peritoneal fluid, a clear rim can be observed at specific TI (**c**)

A multi-TI FAIR-EPI (Fig. 1a) was employed for single-slice perfusion in axial orientation. Sequence parameters were as follows: Constant recovery time TR = 10 s, TE = 12.9 ms, matrix size = 96 × 96, resolution = (625 × 625 × 2000) μm^3^, 14 or 16 TI delays ranging from 100 to 5150 ms, and TA approximately 10 min with respiratory gating. Specific FAIR parameters included a 5 mm-thick slice-selective inversion interleaved with the global inversion. The inversion pulse was delivered by a full passage adiabatic hyperbolic secant pulse with a duration of 16 ms and a bandwidth of 4200 Hz. The slice position was chosen to maximize the liver volume (see Fig. 2).

**Fig. 3:**
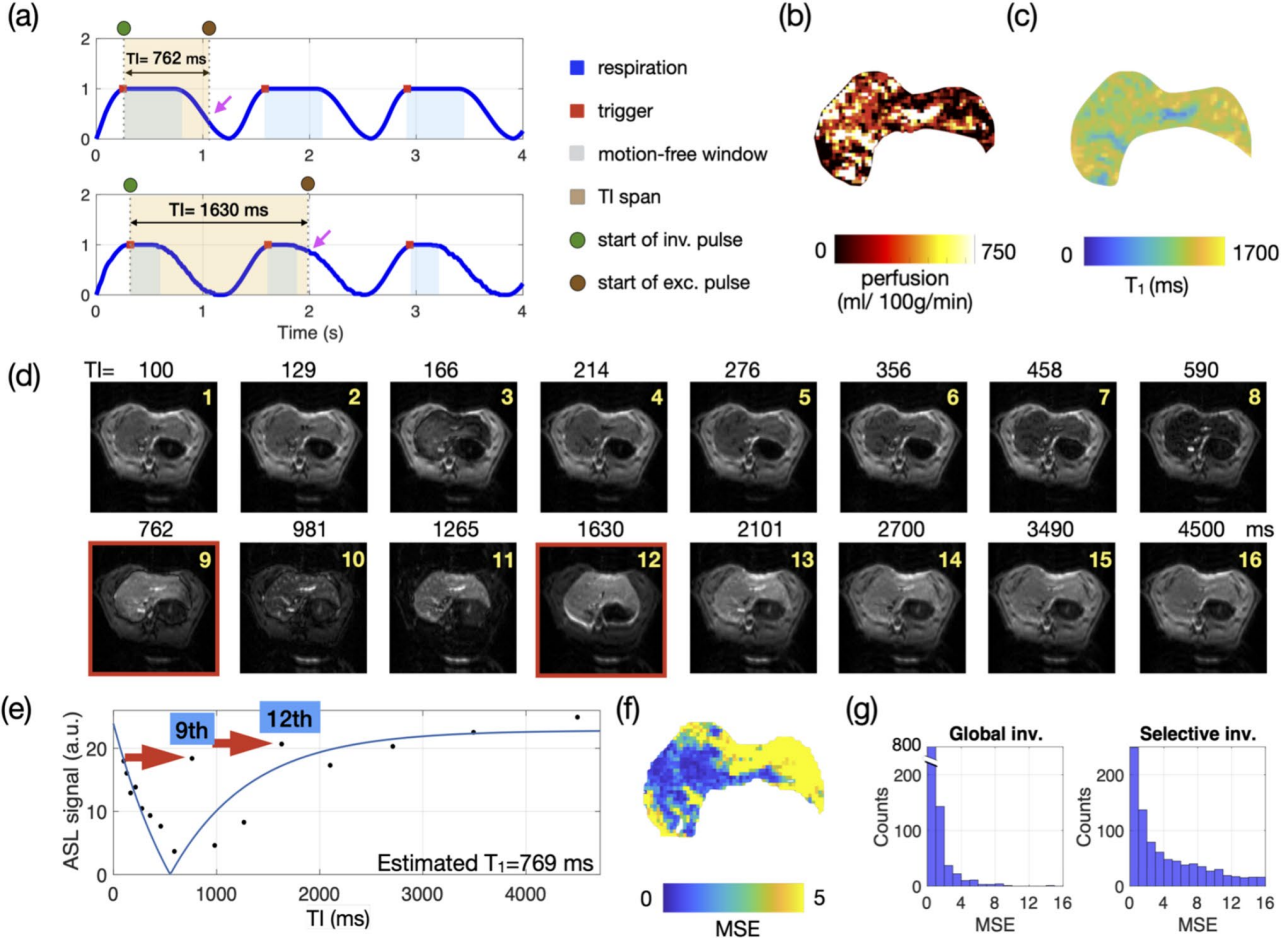
Respiratory modulation in the multi-TI FAIR perfusion MRI acquired in an NMV rat. **a** Schematic demonstration of errors occurring in the respiratory curve of the NMV group: (upper panel) when the inversion starts (green circle) with the TI span (orange shade) coincides with respiratory movement (magenta arrows), the following excitation pulse (brown circle) would not excite the same plane to the inverted plane, resulting inaccurate ASL signal (red boxes in d, red arrows in (**e**). Furthermore, the images obtained in NMV animals are acquired under a mixed imaging window (blue-shaded areas) of regular (upper panel) and irregular breathing (presuming the same respiration rate), which is unsuitable for precise TI planning. **b** Perfusion map and **c** T1 map from selective inversion showed inhomogeneous quantification value in homogeneous liver tissue in a healthy rat. The degree of modulation from the respiration of the inversion recovery ASL image (**d**) and signal curve (**e**) are quantified and visualized in the MSE map (**f**) and MSE histogram (**g**). TI: inversion time, MSE: mean square error, global inv.: global inversion. selective inv.: selective inversion

**Fig. 4:**
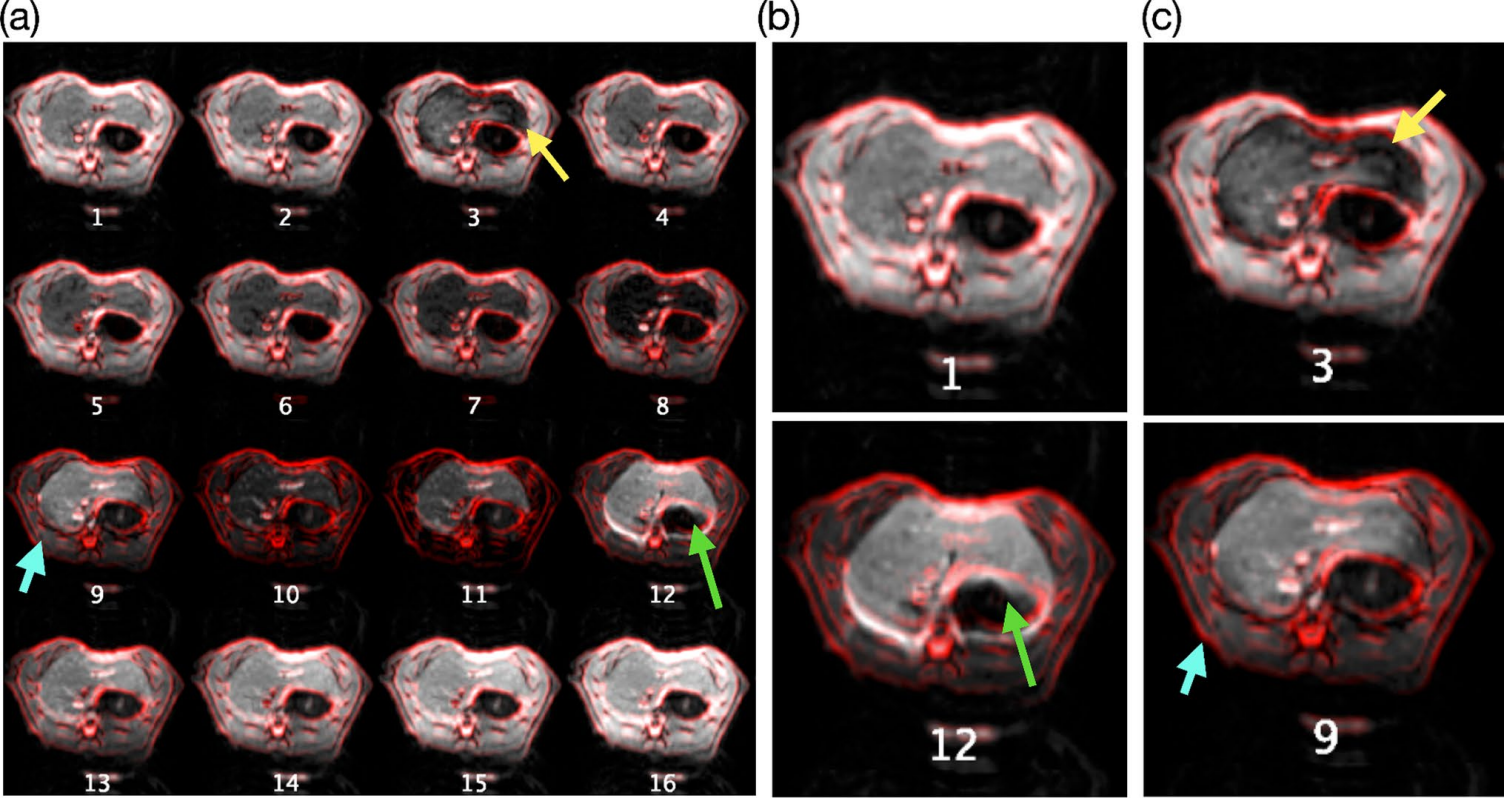
**a** All 16 TI images from the slice-selective inversion acquisition of a non-ventilated FAIR experiment are shown in grayscale, with edge detection from the first TI image overlaid in red contour. A comparison between the edges and the individual images revealed both in-plane motion (green arrows) and through-plane motion (yellow and blue arrows). **b** Enlarged example of in-plane motion: compared to the 1st TI, the smearing observed in the 12th TI image (green arrow) introduces a false signal in the signal void region, leading to signal deviation in the signal recovery curve (Fig. 3e). The elastic liver stretched during inspiration and exceeded the edge contour. **c** Enlarged example of through-plane motion: Compared to the 1st TI, the third TI image showed an inhomogeneous slice profile (yellow arrow). This phenomenon occurs, because the inversion and excitation planes are different due to motion. As a result, respiratory motion prevented the inverted spins from continuing to be excited by the following 90-degree pulse. Likewise, at the 9th TI, the inversion recovery signal curve was expected to be low in signal intensity (cyan); it has a higher signal, indicating that the slice selection inversion and excitation slice are distinct

Instead of the full plateau of end-expiration, the trigger window for gating was shortened to ~ 10 ms and positioned at the onset of the respiration plateau (Fig. 3a) to ensure precise timing for the start of the inversion period. For the NMV group, the TI series was configured as a geometric series [TI_1_ = 100, …, TI_16_ = 4500 ms] (vendor default), designed to provide comprehensive coverage of the signal recovery curve with denser sampling at shorter TIs. Given the non-periodic respiration of NMV group, which could also drift during the scan due to body temperature fluctuations of the animal (e.g., due to SAR heating), the sampling of the recovery curve with the geometrically varying TI times was not pre-calculated to be synchronized with the breathing but provided reasonable coverage even with fluctuations in respiration periods (Fig. 4).

**Fig. 5:**
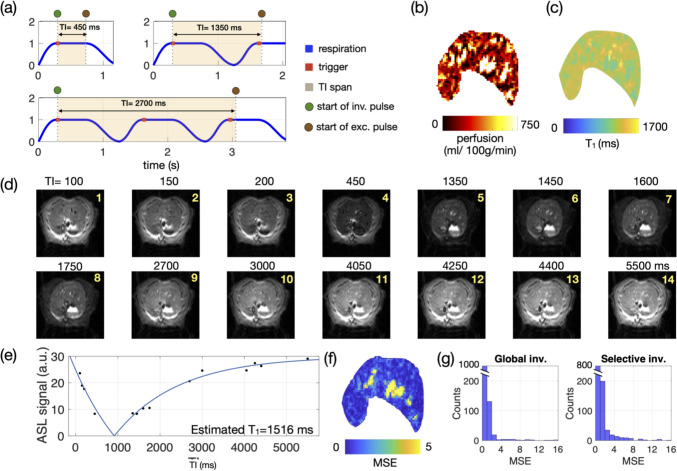
Respiration-aware TI planning in the multi-TI FAIR-based perfusion MRI acquired in an MV rat. **a** The list of TIs was selected to avoid expiration and inspiration in the MV group: When the inversion starts (green circle) with the TI span (orange shade), avoiding coincides with respiratory movement, the following excitation pulse (brown circle) would excite the same/adjacent plane to the inverted plane, resulting an ASL signal that follows the governed equation of inversion recovery signal curve. Furthermore, the images obtained in MV animals are acquired under a controlled condition of regular breathing, which is suitable for precise TI planning. **b** Perfusion map and **c** T_1_ map showed homogeneous quantification values in homogeneous liver tissue in a healthy rat. The degree of modulation from the respiration of the inversion recovery ASL image (**d**) and signal curve (**e**) are quantified and visualized in the MSE map (**f**) and MSE histogram (**g**). The voxels with high fitting errors are primarily located in the surroundings of the vessels, which mainly come from vascular pulsation. From The T_1_ map, the liver parenchyma exhibits comparable T_1_ values. TI: inversion time, MSE: mean square error. Global inv.: global inversion. Selective inv.: selective inversion

For the MV group, the ~ 50 ms acquisition time required for single-slice EPI imaging immediately following the TI period was taken into account, and a list of motion-free TIs was pre-calculated based on the set ventilation rate (see Fig. 5a). This approach ensured that selective inversion and subsequent EPI acquisition were consistently aligned with the same expiratory phase within the respiratory plateau across the different TIs.

### Data analysis

The Bruker macro for ASL evaluation was utilized for a quick online data quality assessment during measurement. For the calculation of the fitting residuals, offline pixel-wise liver perfusion maps were generated in MATLAB (R2019a, MathWorks) by fitting the FAIR image series to the equation

1 $$S_{i} = \left| {S_{0} \left( {1 - 2\alpha e^{{\left( { - TI_{i} /T_{1} } \right)}} } \right)} \right|,$$

where S_i_ represents the measured signal at the corresponding inversion delay TI_i_. The fit parameters included the signal S_0_ at TI = 0 ms, the tissue relaxation time constant T_1_ [27], and the inversion efficiency constrained to $\alpha \in$ [0, 1]. The fitting process was performed using a three-parameter least-square fit (*lsqcurvefit*) to obtain T_1_ maps for both the global and selective inversion FAIR series, T_1,global_ and T_1,selective_, respectively. Liver perfusion maps were then calculated according to [28]

2 $$perfusion = \lambda \cdot \frac{{T_{1global} }}{{T_{1blood} }}\left( {\frac{1}{{T_{1selective} }} - \frac{1}{{T_{1global} }}} \right)$$

A blood tissue partition coefficient $\lambda$ of 0.95 ml/g was assumed for the liver [29], along with an estimated T_1_ value of 2430 ms for blood at 9.4 T [27]. Liver blood flow was then calculated and expressed in units of ml/100 g tissue/min.

To facilitate region of interest (ROI) based analysis, manual segmentation of the liver perfusion maps was performed for the entire liver cross-section and individually for the following lobes: right median lobe (RML), caudate lobe (CL), right lobe (RL), left lateral lobe (LLL), and the left median lobe (LML). The segmentation of the liver lobes was based on the calculated T_1_ map and the contrast of the peritoneal fluid versus liver tissue to depict the rim between different lobes (Fig. 2). Another segmentation was performed for comparison, identifying vessels by visually inspecting and excluding regions with very high perfusion values that formed vessel-like clusters in central anatomical locations.

### Quality assurance

To further evaluate the quality of the measured data, the fit quality of the T_1_ maps was assessed by calculating the mean square error (MSE) for the individual T_1_ fits of each voxel, using the raw residuals r_k_ as

3 $$MSE=\frac{\sum_{k=1}^{n}({r}_{k}{)}^{2}}{n},$$

where n represents the number of different inversion times used in the fit. The squared sum of the residual was obtained from the *resnorm* output parameter of the *lsqcurvefit* function. This fitting procedure was applied to both acquisitions (i.e., global inversion and selective inversion) for each voxel, resulting in a T_1_ map and a residual map of both the selective and the global inversion acquisition.

The edge detection to compare all TI to the first TI was performed using Find Edges in ImageJ and applied to all TI (ImageJ Ver. 1.54 J). The mean perfusion and the mean fitting error were calculated for each region of interest (ROI) to compare the perfusion results between different scan sessions. The mean fitting error was determined as the average mean square error (MSE) values from the T_1_ fit across each ROI. The coefficient of variation (CV) for each ROI was then determined using the mean perfusion (μ) and the standard deviation (σ).

4 $$CV\, = \frac{\sigma }{\mu } \times 100\%$$

## Results

Multi-TI FAIR experiments with approximately 10 min duration have been performed under free breathing (NMV) and ventilated (MV) conditions. In the NMV group, the respiratory period during scan time typically ranged from 1200 to 1500 ms, whereas in the MV group, the period was consistently 1333 ms. This consistency resulted in a wider, usable end-expiration phase—the motion-free interval within the respiratory cycle—of 500–550 ms, accounting for about 40% of the cycle. In contrast, the NMV group presents variable motion-free windows (Fig. 3a, blue-shaded areas), even with the same averaged respiration rate (Fig. 3a, upper and lower panel have identical respiration rate but different I:E ratio).

To optimize data acquisition during the motion-free interval, a shortened trigger window (~ 10 ms) was applied at the beginning of the end-expiration phase (Figs. 3a and 5a, red dot). This approach is highly effective during predictable MV acquisitions, allowing precise timing of the inversion pulse and enabling accurate planning of TIs even several respiratory cycles later (e.g., TI = 5500 ms). Typically, the trigger window for rapid sequences is adjusted to match the motion-free period of respiration for optimal scan efficiency. However, for multi-TI perfusion scans that span multiple respiratory cycles and use long repetition times, maintaining a short trigger window ensures a well-defined inversion time point and consistent motion-free intervals for different TIs. Consequently, the excitation pulse and EPI acquisition were consistently aligned with the end-expiratory phase. Using MV and a short, early trigger, this acquisition scheme improved acquisition consistency, as demonstrated by the clearer signal profiles across images for different TIs (compare Figs. 1c and d).

The perfusion results using a short trigger window acquired from a free-breathing animal are shown in Fig. 3. Figure 3d shows the original EPI images at all TIs with selective inversion pulses. The visible liver was segmented as ROI, which is the area shown in Fig. 3b (perfusion map) and Fig. 3c (T_1_ map from the selective inversion). The signal recovery of the single voxel at liver parenchyma avoiding vessels is shown in Fig. 3e, where aberrant ASL signals at the 9th and 12th inversion time TI were observed in the NMV animal (Fig. 3d, e). These aberrations resulted in poor fit in perfusion and *T*_1_-relaxation time (Fig. 3c), which are reflected by the high values in the MSE map (Fig. 3f, yellow area upper right) and histogram (Fig. 3g). Since the normal liver is a parenchymal organ with a homogeneous appearance, substantial perfusion differences between liver lobes were not observed in these healthy rats.

All EPI images were visually inspected for motion artifacts to investigate the source of the aberrant ASL signals observed in the 9th and 12th TI. This was done by applying an edge-enhanced red-colored overlay of the first TI image onto the remaining images for comparison (Fig. 4a). Due to the inherent motion insensitivity of EPI, noticeable aliasing or ghosting artifacts were rarely observed. Upon closer inspection, smearing artifacts due to in-plane motion up to 6 pixels (Fig. 4b, green arrow) and inhomogeneous excitation (Fig. 4c, yellow arrow) were observed in different TI images. Additionally, through-plane motion (Fig. 4c, cyan arrow) specifically affected the 9th TI, where the liver tissue was expected to appear dark. Collectively, these motion artifacts contributed to inaccuracies in perfusion quantification across the lobes.

For the MV group, no obvious deviating ASL signal (Fig. 5d, e) was observed. The fitting error shown in the MSE map (Fig. 5f) and the histogram (Fig. 5g) was reduced throughout the segmented liver area. In the montage (Supplementary Fig. 1), comparing all the TI images to the 1st TI, a minimal in-plane smearing artifact can be observed in the surrounding area of vessels of the portal vein (Supplementary Fig. 1, cyan arrow), the hepatic vein (yellow arrows), and the aorta (green arrows), respectively. These are most likely small remaining motions caused by blood pulsation, whereas respiration motion has been largely suppressed.

**Fig. 6:**
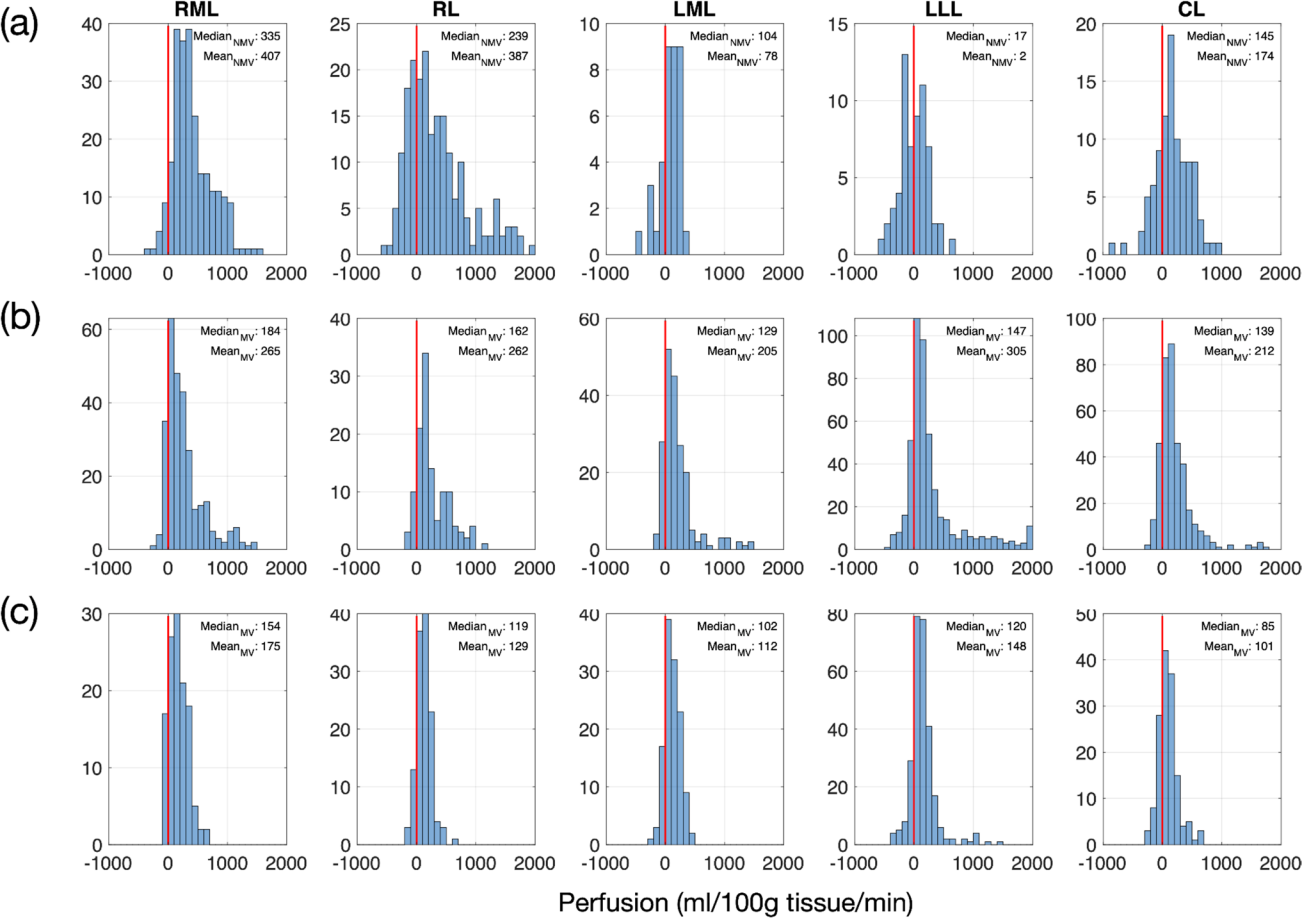
Comparison of perfusion for different conditions. **a** NMV including all voxels resulted in a broad histogram and falsely low values for median and mean; in this set, especially in the LLL with mean of 2ml/100g tissue/min **b** MV resulted in the narrow, biphasic histogram, with the second peak representing the vessels in the liver lobes, in the LLL now has a mean of 305ml/100g tissue/min **c** MV after excluding the vessels resulted in a narrow monophasic peak in the LLL, with mean perfusion of 148ml/100g tissue/min. Overall, perfusion in NMV (**a**) demonstrated a broad spread of histogram distribution with a tapering tail, indicating high data variability. In contrast, MV (**b**) demonstrated a more concentrated histogram distribution with narrowed tails, which was further eliminated when excluding vessels (**c**)

A representative histogram comparison between the NMV and MV groups revealed a broader distribution in the NMV group (Fig. 6a, FWHM = 482–493/ NMV animal) compared to a narrower, more distinct biphasic distribution in the MV group (Fig. 6b, FWHM = 271–381/ MV animal). The coefficient of variance in the NMV group was consistently higher (144–706% /NMV animal) than in the MV group (62–90%/ NMV animal), reflecting higher signal variability. MV increased the mean perfusion of the LLL from the NMV of 2 ml/100g tissue/min (Fig. 6a) to 305 ml/100g tissue/min (Fig. 6b), due to the reduced negative perfusion from poor fit. As a result, the mean perfusion in LLL aligned with the perfusion in the other lobes; MV effectively enhanced the reliability of the perfusion measurements. Despite the similar mean values in RL under NMV and MV conditions, the histogram distribution is broader in NMV compared to MV, suggesting less precision in NMV. Tissue-only perfusion values were isolated by removing the blood vessels from the segmentation, and the biphasic distribution (Fig. 6b) became a monophasic distribution (Fig. 6c). The calculated mean perfusion of tissue without the larger vessels was consequently lower by approximately a factor of 1.5–2, depending on how many vessel voxels relative to the entire lobe ROI were contained in that slice and have been removed.

In a preliminary investigation on the effect of data exclusion in perfusion quantification on the same animal (Supplementary Fig. 2), aberrant data points were excluded for a better fitting result, and the accuracy of the perfusion maps (Supplementary Fig. 2a) was improved. The MSE of the T_1_ maps was reduced (Supplementary Fig. 2b), and the perfusion distribution was shifted rightward in the lobe of RL, LML, LLL, and CL (Supplementary Fig. 2c, blue: complete TI series, red: data exclusion). However, the spread of the histogram remained relatively constant following data exclusion compared to using the full set of TI values, indicating that data exclusion only affects the accuracy rather than the precision of the perfusion data.

**Fig. 7:**
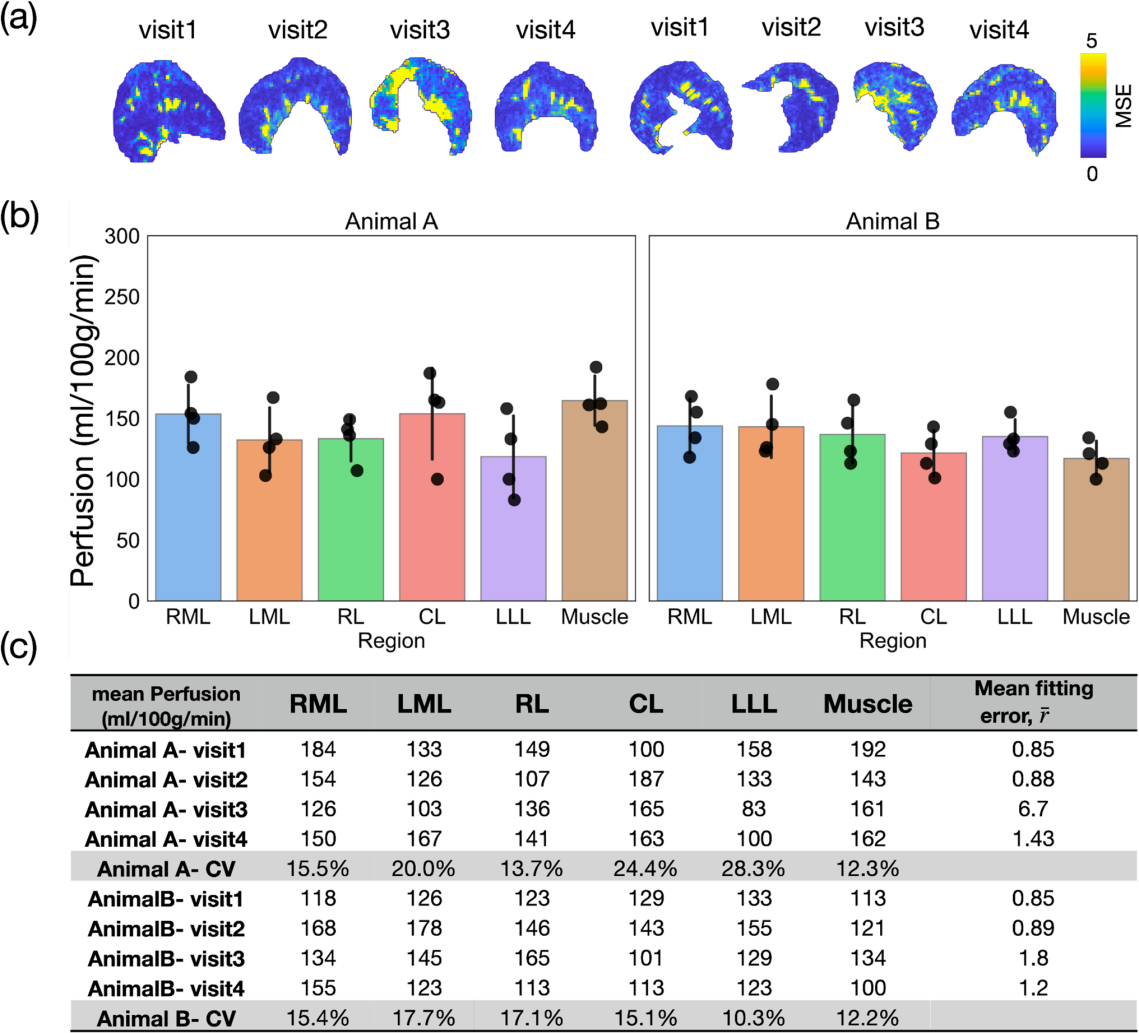
Repetitive evaluation of liver perfusion per lobe in MV rats (n = 2). **a** The map of the MSE demonstrates the fitting quality throughout five lobes in four visits of perfusion assessments. The high fitting residual areas coincide with the location of the major vessels due to pulsation. In visit 3 of animal A, part of the voxels had been affected by the movement of the intestine due to digestion (e.g., peristalsis), resulting in a high mean fitting residual ¯r. **b** The estimated perfusion lies between 100 and 180 ml/min/100g tissue, excluding all major vessels. Error bar: standard deviation. **c** The repetitive perfusion estimation showed a ~ 20% coefficient of variation through the five lobes in four visits. *CV* coefficient of variance

To assess the reproducibility of perfusion measurements with MV, two animals repetitively underwent multi-TI FAIR ASL perfusion (n = 4 sessions per animal), and the perfusion results and their variability were evaluated. No apparent trends within or between animal variations were observed. The perfusion results throughout the segmented five liver lobes from each session revealed a range of 100–185 ml/100g tissue/min in repeated measurements, excluding the high perfusion voxels contributed by vessels (Fig. 7b, c). The mean perfusion for RML, LML, RL, CL, and LLL lobes were 149 $\pm$ 8, 137 $\pm$ 9, 135 $\pm$ 7, 137 $\pm$ 11, and 126 $\pm$ 9 ml/100g tissue/min, respectively (mean $\pm$ S.E.M.). The within-session CV ranged from 5–22% and between-session CV 12–20%, neglecting session 3 in animal A. In that session, part of the image in the TI series had been affected by the movement of the intestine due to digestion (e.g., peristalsis). The peristalsis motion is detectable and reflected in a high MSE (Fig. 7a, yellow) and the mean fitting error (Fig. 7c), which could be utilized to decide on a re-scan for improved accuracy during the session. In addition, perfusion segmentation of the muscle provides a comparative baseline for perfusion variability. The muscle perfusion values serve as an intra-session reference outside the liver, which exhibits low perfusion variability. Interestingly, the perfusion of liver parenchyma, excluding large vessels, here has a similar perfusion value to that of the muscle, which might be due to the vasodilative effects of isoflurane.

## Discussion

ASL perfusion is based on the difference between two images or, in the case of multi-TI FAIR, two T_1_ maps: one where incoming blood to the imaging slice is labeled and a control image where no labeling occurs. The resulting difference between the label and the control is approximately less than 1% [4, 13]. Given this small signal change, accounting for confounding factors such as respiratory motion artifacts is essential to ensure accurate quantification. This is particularly important when measuring perfusion with multi-TI inversion recovery in the liver, as sufficient time must be invested to adequately sample the entire T_1_-relaxation curve and accurately determine the T_1_-relaxation time constant. A special attention must be given to ensure that respiratory motion does not interfere with the longer TI measurements, as any motion during the acquisition periods can distort the inversion recovery signal and compromise the accuracy of the T_1_ maps and, therefore, the perfusion data. While EPI is relatively motion-insensitive and can produce adequate images, motion-related distortions can still compromise the accuracy of perfusion quantification. A significant source of error in ASL signal measurement occurs when the inversion slice and the imaging slice are misaligned due to through-plane motion. This study aimed to develop and evaluate an experimental protocol for achieving reliable and reproducible liver perfusion quantification in rats. We consider mechanical, predictable ventilation to be crucial for achieving reliable and predictable results, as only strictly regular respiration allows us to plan the time points for long TIs up to 5500 ms, i.e., up to 5 respiration phases “into the future” after the trigger. Predictable and reproducible trigger points are also further facilitated using a short trigger window at the beginning of each motion-free phase.

A carefully controlled anesthesia regime supported the predictable respiratory movement in the MV group. In this study, isoflurane was administered at a concentration of 3%, using oxygen-enriched air. This was combined with optimized ventilation tubing to minimize dead space and exhalation resistance. Continuous monitoring of respiration rate and end-tidal CO₂ levels ensured stability throughout the imaging procedure. Good lung compliance and complete suppression of spontaneous breathing also required adjustment of the tidal volume for ventilation.

Intubation and mechanical ventilation, widely utilized in rats and larger rodents, can also be effectively adapted for use in mice [30, 31]. However, while readily available clinical materials such as 14-gauge cannulae with Luer connectors are often used as endotracheal tubes for intubation in rats, the significantly smaller size of mice adds additional complexity. In mice, the endotracheal cannula must be of an appropriate size, typically in the range of 20-22G [30, 31] (equivalent to an outer diameter of 0.7–0.9 mm). The total dead space—the volume from the Y-branch connecting the inspiratory and expiratory tubing to the distal end of the tube inside the trachea—must be significantly smaller than the tidal volume of the mouse, which is approximately 0.6 mL in a 20 g ventilated mouse [31]. An excessive dead volume can severely reduce the efficiency of gas exchange at the alveoli and lead to hypercapnia. Importantly, the classical method relies on the water replacement method to measure V_T_ (0.16–0.2 mL for mice [32]), whereas Vt on the mechanical ventilator is measured using a flow sensor, which inevitably has the effect of dead space and therefore operates at a higher value to ensure sufficient gas exchange at the alveoli. In addition, using Q-tips to gently wipe off saliva near the larynx prior to cannula insertion can effectively prevent introducing mucosal bacteria to the lung due to capillary action, which is more pronounced in small-diameter cannula. Adapting these processes can ensure successful ventilation in both mice and rats while maintaining their physiological stability during experimental procedures.

Since ventilation poses the risk of lung damage, we carefully optimized the V_T_ and respiratory rate (breaths per minute) in the intubated rats. Initially, we set the V_T_ to 7 ml/kg, later increasing it to 10 ml/kg [22] to ensure adequate gas exchange while preventing spontaneous breathing. Notably, although a V_T_ range of 6–10 ml/kg is generally considered safe [33, 34], studies have shown that rats mechanically ventilated with a V_T_ of 12 ml/kg exhibit significantly higher neutrophil infiltration and reduced septal volume density, indicating inflammation and compromised tissue perfusion [34]. At higher tidal volumes, such as 22 ml/kg, epithelial and endothelial cell damage has been reported [35].

In contrast to the negative pressure in the lungs during spontaneous breathing, the positive pressure that enters the lungs from the ventilator can compress the heart and large vein in the thorax during inspiration. This compression can impede venous return to the heart and lead to a drop in blood pressure [23], which could alter perfusion. Therefore, the duration of inspiration should be as short as possible. However, when testing an I:E ratio of 1:3, the inspiratory time was too short, resulting in insufficient oxygenation and lung recruitment, which ultimately led to additional spontaneous breathing in the animal. Therefore, the final I:E ratio was set to be 1:2 to achieve a sufficiently short inspiration and an exhalation state with as little movement as possible.

Intubation and ventilation of the animals are associated with stress and can cause airway injuries. To minimize invasiveness due to ventilation while addressing the issue of unpredictable respiratory motion after long inversion times, two trigger points could also be incorporated in the sequence, one for the inversion and one for the imaging part. To compensate for the randomly longer TIs due to the extra trigger delays, the actual timing would have to be recorded either by the scanner or, e.g., by external data loggers. The recorded timing could then be used to calculate the actual TIs when fitting the two FAIR T_1_ maps. Since the two maps are calculated individually, slightly different TIs in the selective and global T_1_ maps should not cause any problems as long as the sampling pattern is still dense enough to represent inversion recovery.

In addition to respiratory motion, multiple other motion sources, including heart beat, gastrointestinal peristaltic movements, challenge liver imaging. Even with a perfect respiratory synchronization, slight motion between FAIR images can still be present [36]. For example, the stomach is sometimes almost embedded between the liver lobes. It can cause irregular motion due to liver displacement and non-linear deformations, leading to an inhomogeneous slice profile or mismatches between inversion and imaging slices. In-plane motion can result in image blurring and misalignment between different TIs or between global and selective acquisitions. However, these other sources of motion are comparatively small in terms of respiratory motion and typically affect liver areas close to the stomach, heart, or bowels. If high perfusion accuracy across the whole liver is required, the study protocol could be extended to prior fasting and bowel motion-reducing drugs, e.g., application of muscarinic acetylcholine antagonists, such as atropine, which effectively reduces peristaltic contraction and excessive salivary secretion, avoiding backflow and choking the animals [37].

There are several approaches to mitigate/minimize abdominal motion during data acquisition in preclinical MRI. Bilreiro and colleagues [38] tested the dosage of administration of hyoscine butylbromide (BUSC), an anticholinergic medication used in the clinic to treat abdominal pain, to suppress bowl movement in the mice. They found that intraperitoneal injection of BUSC (5mg/kg) can effectively reduce the bowel movement for up to 45 min. Moreover, administration of BUSC is more effective than food deprivation for 4.5–6.5 h in reducing bowel movements, providing an alternative while applying for animal regulatory approval. Notably, BUSC has the side effect of increasing heart rate, which may consequently alter the liver and renal perfusion, requiring attention for cross-comparison with other perfusion studies. Zhao and colleagues [39] avoided respiratory motion in renal ASL by pharmacologically minimizing peristalsis and reducing the respiration rate. They used a fixed TI and fast EPI readout to squeeze in the repetition in every other respiration cycle. With this approach, the reproducibility of the ASL data was high. However, it is limited within a single TI ASL approach, and (hence) the T_1_ of the tissue relies on literature values, which may be changed in disease conditions such as neuropathy [40] and allograft [41]. In addition to the fairly robust FAIR perfusion sequence, pCASL is a promising ASL technique that would allow assessment of additional timing information due to the variable post-labeling delay [42–44] and has been implemented for abdominal imaging [45–47]. However, the inversion efficiency depends on the flow in the labeling plane and has to be carefully optimized [48, 49].

There are few attempts to target respiratory and cardiac motion simultaneously. Simply adding an ECG trigger on top of the respiratory trigger will only correctly trigger the inversion, but the imaging after a specific inversion time will fall anywhere within the ECG period since it is not predictable enough. Campbell-Washburn and colleagues [9] successfully dealt with respiratory motion in the heart of mice. They implemented a segmented sequence and a data logger that enabled synchronization and recording of ECG peaks, respiration profiles, and RF pulses for offline screening. The techniques were later applied to the imaging of rodent liver [10, 11]. However, their approach collects an oversampled k space and replaces the corrupted k-space offline, which extends acquisition time. It also requires a dedicated implementation and integration between the sequence and reconstruction. Our current study aims to raise awareness of respiratory modulation in a multi-TI FAIR-based ASL study and offers solutions for a robust and reproducible liver perfusion MRI.

In addition to care in data acquisition, data quality assessment is equally important, especially for NMV acquisitions. As shown in Supplementary Fig. 2, the detection and exclusion of corrupted data points improves the quality of the perfusion map. However, this also requires careful detection and selection of corrupted data points. Once the perfusion data are collected, it is essential to inspect (1) the image pair (i.e., the labeled and control image) per TI whether there is a sudden jump in image intensity; (2) the image intensity profile over TI: whether the overall image intensity follows the magnetization nulling and grows again under T_1_ relaxation. Also, selective inversion is more likely to be contaminated by bowel motion, and the global inversion is more likely to be affected by B_1_ inhomogeneity [50]. Monitoring both the signal variations in the whole liver and the FAIR series could elicit an immediate reaction for further acquisition. The ASL perfusion data acquired under MV also corrected the mean perfusion value of LLL from 2 to 305 ml/100g tissue/min, which is in line with the reported data in in vivo MRI [11] and isolated rat liver [51].

The MSE is a convenient measure that can be derived for all T_1_ fits for both the selective and global T_1_ maps and is a practical and sensitive quality criterion for the derived perfusion map. Least-squares fitting, in our case specifically MATLAB’s *lsqcurvefit,* is one of the most commonly used fitting algorithms for ASL that also allows for constraints while minimizing the deviation of the data from the fitted curve. Its trust-region method allows the setting of lower and upper boundary conditions to enforce valid parameter ranges and provides faster convergence to a global minimum [36]. Since the performance of fitting algorithms depends at least to some degree on the initial values, using the signal zero crossing $T{I}_{null}={T}_{1}ln(2)$ could help to better define the initial value, leading to a more accurate quantification of T_1_ [52].

Other methods improve the data quality after image acquisition: non-rigid body registration [53, 54], the Elastix package [55], and data exclusion (Supplementary Fig. 2) are practical tools for better fitting quality, but they may not fully address motion issues. Because co-registration algorithms often rely on image intensity changes to detect motion, they may misinterpret the natural signal fluctuations in the inversion recovery as motion. In the case of through-plane motion, the image is contaminated with corrupted inversion information that cannot be recovered, making co-registration even less effective. Instead, combining MV to limit through-plane motion with a robust in-plane co-registration algorithm could provide a more effective solution for respiratory and other types of motion in liver imaging.

To date, DCE-MRI is the most commonly used technique for assessing liver perfusion. However, ASL-MRI is a more suitable alternative for studies in which perfusion is assessed at consecutive time points, as it avoids the need for contrast agent administration and its clearance. Our work presents a straightforward and easy-to-use quality assurance protocol for ASL-MRI of the liver with multi-TI inversion recovery. The data highlight the importance of acquiring artifact-free images by actively synchronizing the respiratory motion using mechanical ventilation and coordinating TI planning with a shortened trigger. For immediate quality control, to maintain experimental stability and to detect outliers, we strongly recommend the visualization of the fitting residuals map as a critical quality control procedure when coordinating the TI list for ASL perfusion at the acquisition stage. The proposed protocol offers a comprehensive approach for performing multi-TI inversion recovery for reliable and reproducible liver perfusion in rats, enabling researchers to achieve consistent results.

## Supplementary Information

Below is the link to the electronic supplementary material.

Supplementary file1 (DOCX 3188 KB)

## Data Availability

The data supporting this study's findings are available from the corresponding author upon reasonable request.
