# Supplementary material for: A quality assurance protocol for reliable and reproducible multi-TI arterial spin labeling perfusion imaging in rat livers
Source: MAGMA. 2025 Jan 4;38(3):503–17. doi: 10.1007/s10334-024-01223-1 (PMC12255561; doi:10.1007/s10334-024-01223-1)
Supplement: Supplementary file 1 — Supplementary file1 (DOCX 3188 KB) [file 10334_2024_1223_MOESM1_ESM.docx]

**Supplementary materials**

**
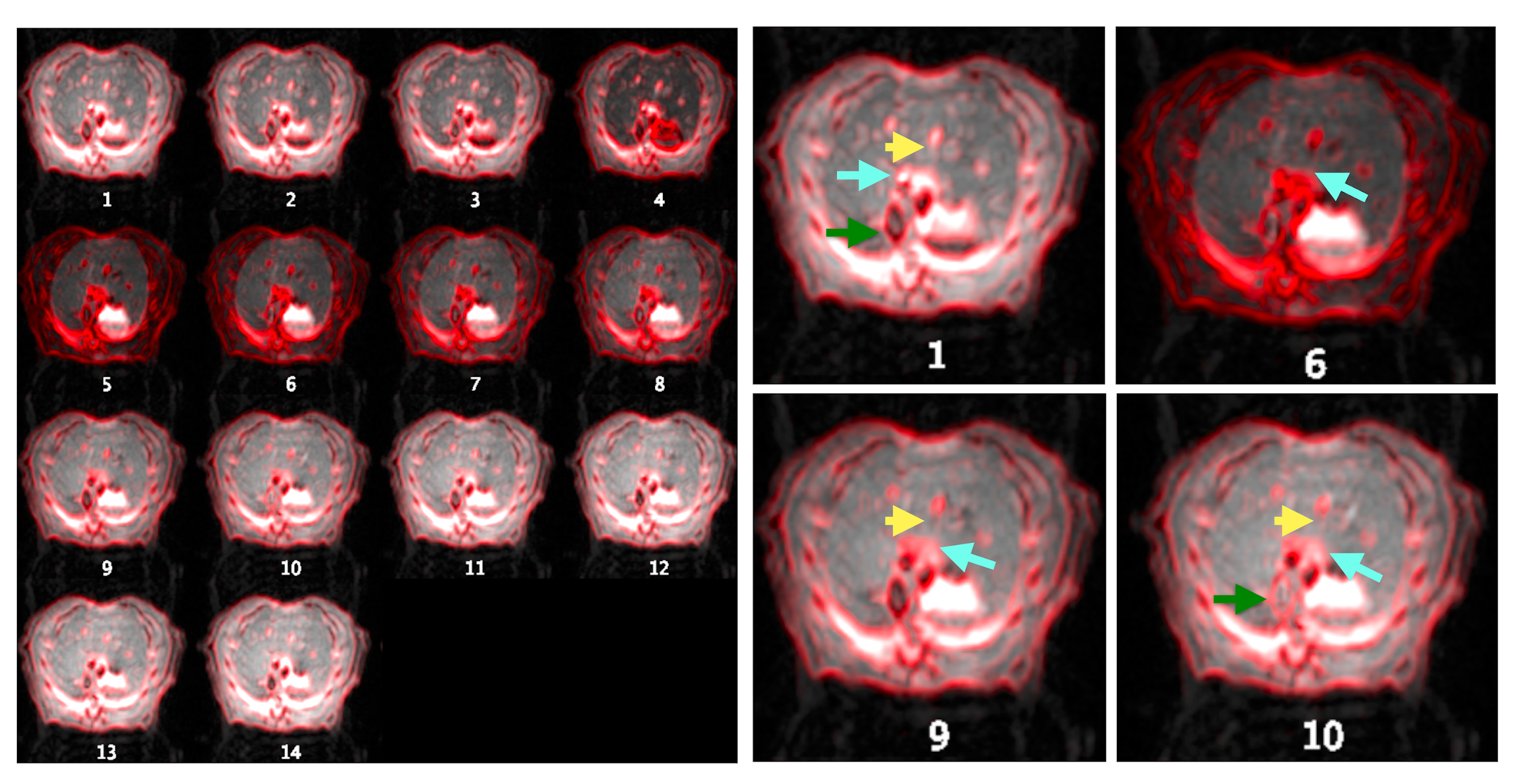
**

**Supplementary Figure S1**: Montage of all 14 images at different TIs of the slice selective inversion acquisition of a mechanically ventilated FAIR experiment is shown. The images are in grayscale, overlaid by a red-colored edge detection from the first TI. MV minimized respiration-induced motion. Example enlarged case: Compared to the 1st TI, the in-plane motion due to the stretching of the liver was shown in the surrounding area of the aorta (green arrows), the portal vein (cyan arrows), and the hepatic vein (yellow arrows). No apparent through-plane motion was observed.

**
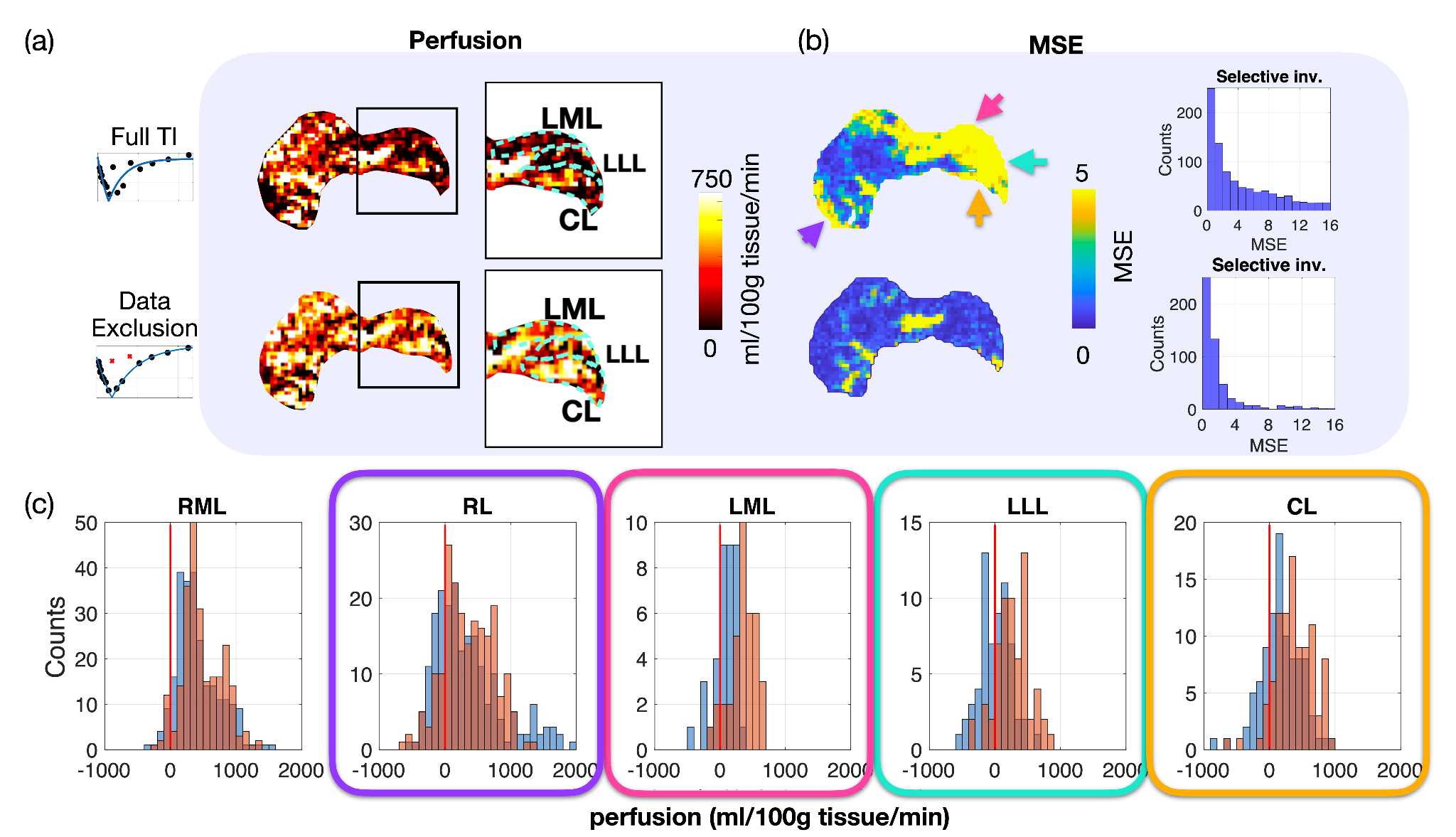
Supplementary Figure S2**. Preliminary test on the effect of data exclusion in multi-TI FAIR in quantification of perfusion and fitting residual maps in the same healthy rat. (a) The perfusion map using the full TI (upper row) showed a tendency of hypoperfusion in the LML, LLL, and CL, which were corrected by data exclusion (lower row). (b) The fitting residual map using full TI was likewise replenished by data truncation, resulting in a decent fitting quality throughout each lobe. (c) Quantitative comparison of pixel-wise perfusion between the full TI(blue histogram) and data truncation (red histogram) showed a median shift from 17-328 ml/min/100g tissue to 252-406 ml/100g tissue/min. The latter quantification result agrees with the findings from the literature [11]


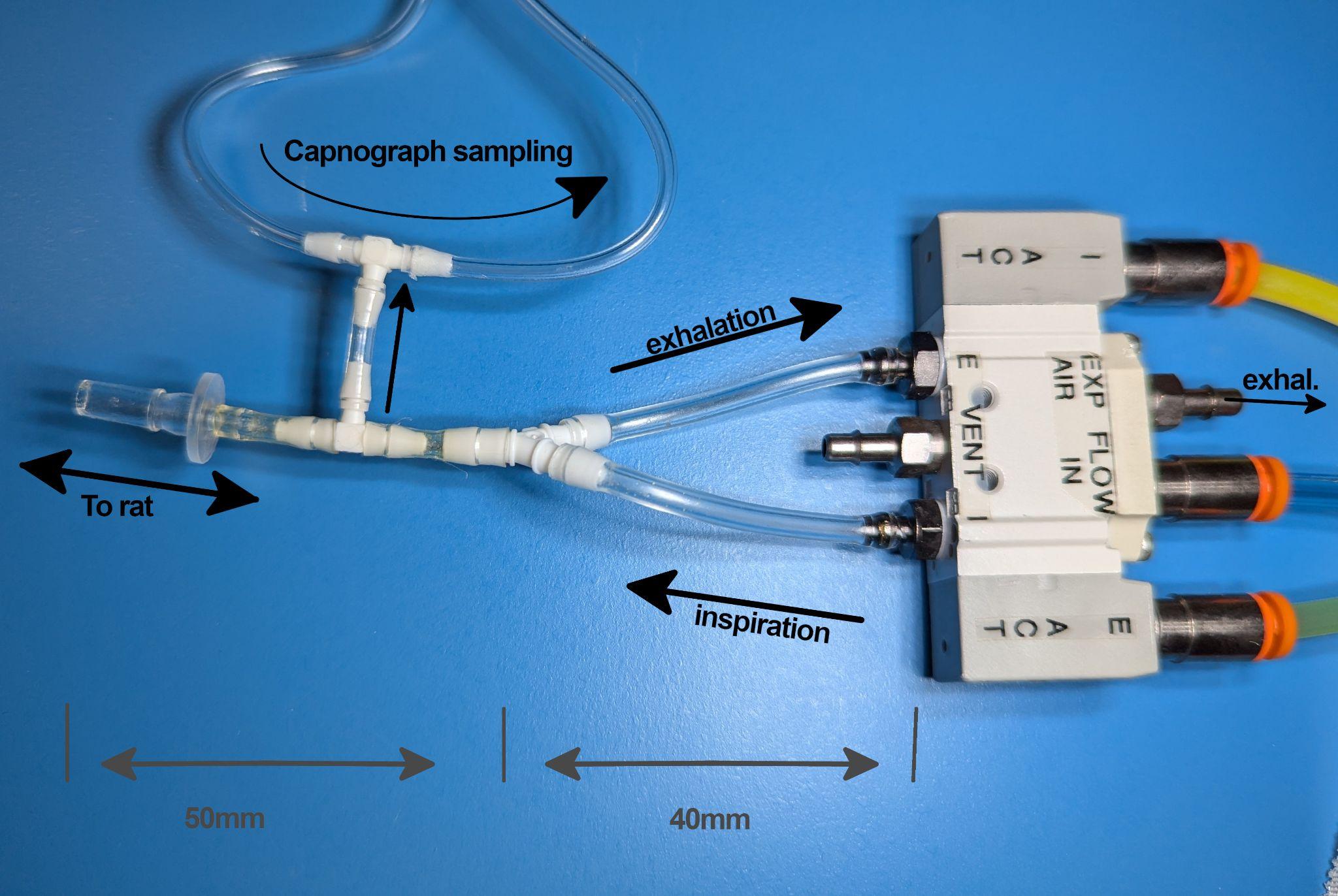


**Supplementary Figure S3:** Ventilation setup used with the MRI-1 Ventilator (CWE Inc.). The ventilator, which is not MRI-compatible, was positioned outside the magnetic stray field. MRI-compatible valves on the right side were controlled by compressed air, switching between inhalation and exhalation. The distance from the Y-connector to the animal represents the "dead volume"—the air that moves back and forth during respiration—and is minimized as much as possible while allowing gas sampling via a T-connector for capnography. To further reduce exhalation resistance, the tubing between the valve and the Y-connector was shortened to 40 mm, and 1.6 mm inner diameter (ID) tubing was used. This required positioning the valves close to the animal's head.
